# Excessive and Problematic Internet Use During the Coronavirus Disease 2019 School Closure: Comparison Between Japanese Youth With and Without Autism Spectrum Disorder

**DOI:** 10.3389/fpubh.2020.609347

**Published:** 2020-12-17

**Authors:** Kentaro Kawabe, Rie Hosokawa, Kiwamu Nakachi, Ayumi Yoshino, Fumie Horiuchi, Shu-ichi Ueno

**Affiliations:** ^1^Department of Neuropsychiatry, Ehime University Graduate School of Medicine, Toon, Japan; ^2^Center for Child Health, Behavior and Development, Ehime University Hospital, Toon, Japan

**Keywords:** COVID-19, internet addiction, autism spectrum disorder, children, problematic internet use

## Abstract

Internet use in the youth has increased manifold during the coronavirus disease 2019 (COVID-19) pandemic. Individuals with autism spectrum disorder (ASD) generally have a higher risk of problematic internet use. The aim of this study is to investigate the differences in internet and related digital media use between children with ASD and their typically developing counterparts during the COVID-19 pandemic. In this online survey in Japan conducted from April 30 to May 8, 2020, we analyzed digital media time of 84 children with ASD and 361 age- and gender-matched controls before and after school closure. Digital media use duration was significantly longer in the ASD group than in the control group before the pandemic. The increase of media use time was more prominent in the control group than in the ASD group. We observed excessive Internet use among children with ASD and without ASD, especially during the COVID-19 pandemic. It is necessary to establish strategies to prevent excessive internet use in not only children and adolescents with ASD but also without ASD in the post-pandemic world.

## Introduction

Coronavirus disease 2019 (COVID-19) infection is evolving rapidly, with an increase in the number of reported cases and affected countries worldwide (1). The World Health Organization declared the COVID-19 outbreak a public health emergency of international concern on January 30, 2020 and a pandemic on March 11 (2). In view of the rapid increase in COVID-19 cases from the end of February, the Japanese government declared the closure of elementary and junior high schools from the 1st through 12th grades on March 2 and a public health emergency of international concern on April 7.

The closure of schools and other educational facilities poses a significant disruption to daily life and is a source of stress for children and their families. In response to the crisis, governments in Japan have introduced a series of steps aimed at curbing the effects of the pandemic, such as maintaining social distance (a minimum of 2 m) and the temporary closure of cultural and entertainment facilities. As a result, children's interactions, both physical and intellectual, with their peers have reduced, which may further induce social isolation and loneliness. With regard to managing this situation, information and communications technology (ICT) holds promise, as through its use, children can continue to engage in educational and entertainment activities, stay in touch with friends using social networking services, and access entertainment or educational content, all while maintaining social distancing. ICT can alleviate social isolation through the development of a sense of connection, maintenance of existing relationships, facilitation of social support, engagement in activities of interest, and enhancement of self-confidence (3).

Although ICT is proving to be an important tool during the COVID-19 pandemic, there are concerns about the rise in problematic internet use and internet addiction among the youth. In a Japanese survey of 8,464 junior high school to university students conducted between March 27 and April 6, during the pandemic, over 80% of the participants were spending more time on YouTube than before, while 40–50% were also spending more time on gaming apps (4). Autism spectrum disorder (ASD) is characterized by difficulties in reciprocal social interaction skills; deficits in communication skills; stereotypic, obsessive, or repetitive behaviors; and restricted patterns of interests and activities (5). In general, adolescents with ASD tend to devote themselves to video games or internet use. Adolescents with ASD who also have attention deficit hyperactivity disorder symptoms have a higher risk of internet addiction (6). Owing to the characteristics of ASD, it can be difficult for children with this condition to understand the context of school closure and manage their internet use time at home during the COVID-19 pandemic (7). Adolescents with ASD have been identified as a high-risk group for complications in mental health from COVID-19 (8). To our knowledge, there are no studies about internet and digital media use in adolescents with ASD during the COVID-19 pandemic. Thus, we hypothesized that internet use in children and adolescents with ASD differs from that in their typically developing counterparts during the COVID-19 pandemic. The objective of this study was to explore the difference in internet and digital media use between children and adolescents with and without ASD and compare the change in use time in these groups before and during the COVID-19 pandemic.

## Methods

### Participants

This cross-sectional and matched case-control study was conducted online from April 30 to May 8, 2020, during the period of school closure in Japan. Members of the ASD group were outpatients at Ehime University Hospital, Matsuyama Kinen Hospital, and Horie Hospital in Ehime prefecture. Matsuyama Kinen hospital and Horie hospital were psychiatric hospitals. These hospitals have specialized psychiatry outpatient clinic for children and adolescents. The inclusion criteria for children and adolescents were: [1] aged 6–18 years; [2] diagnosis of ASD based on the Autism Diagnostic Observation Schedule-2, Autism Diagnostic Interview-Revised, or Diagnostic and Statistical Manual of Mental Disorders-5 criteria; [3] attending elementary, junior high, or high school; [4] residing in Ehime prefecture; and [5] provision of written informed consent by their mothers. The control participants were invited to this survey through social media. The inclusion criteria for children and adolescents were: [1] aged 6–18 years; [2] no history of visiting hospitals regarding a child's development; [3] attending elementary, junior high, or high school; [4] residing in Ehime prefecture; and [5] provision of written informed consent by their mothers. The participants were recruited through snowball sampling.

### Procedure

Mothers whose children met the inclusion criteria were invited to participate in the online survey using the Google Forms software in Japanese. The link to the questionnaire was sent via a letter in the ASD group and social media in the control group. The social media was used LINE, which was first released in 2011 and then became very popular messaging and social media system in Japan. Upon receiving and clicking the link, participants were automatically transferred to the page providing information about the study.

### Instruments

The online survey included three categories: (a) demographic data including age, gender, and school level (elementary school: ages 6–12, junior high school: ages 12–15, and high school: ages 15–18; (b) three yes-no questions: “Is your child stressed by the COVID-19 pandemic?” “Is your child making fewer visits to the after school activities, e.g., lessons, culture schools, education centers, and rehabilitation centers?” After school activities are provided by private agency or establishment. It was not part of school life and Japanese government did not declare the closure of after school activities, therefore if participants want to utilize after school activities, they can access during the school closure period. Another yes-no questions: “Are you spending more time playing games with your child since school closure?”; and (c) multiple choice questions related to digital media use time, “How long did your child spend using the internet or digital media use on weekends before school closure?” and “How many hours a day is your child spending on the internet or digital media use on weekends during the COVID-19 pandemic?” The response options were from 0 min to 15 h, and every 30 min.

### Data Analysis

In this study, we planned to recruit about 125 ASD participants and about 500 participants as the control group. The sample size was calculated on the basis of two-sample *t*-tests using G*Power 3.1.9.2 software (9). An effect size of 0.5, a significance level of α = 0.05, a statistical power of 1-β = 0.95, and a 1:4 allocation ratio between the ASD and control groups were also considered. Sample size calculation was performed before initiating recruitment. Descriptive statistics were used to describe the distributions of the participants' characteristics. The results were expressed as median (25 and 75% quartile) for continuous variables and percentages for categorical variables. The Mann-Whitney *U*-test was used for the comparison of numerical variables. The chi-square test was used for the comparison of categorical variables, and to compare responses between the two groups. The Wilcoxon signed-rank test was used to compare the change in internet or digital media use time before and during the pandemic. All tests were two sided, and the significance level was set at 5%. All data were analyzed using SPSS version 22.0 (IBM Corp., Armonk, NY, USA) for Windows and R version 3.6.3.

### Ethics

Data were protected according to the General Data Protection Regulation. The text message bearing the link to the Google Form that was shared with the participants contained the title of the study, its aim, eligibility for participation, potential advantages and disadvantages of participation, and the average time required to answer all questions, which was 5 min. The questionnaire was anonymized. In addition, the first page of the Google Form mentioned the informed consent requirement.

## Results

### Characteristics of the Study Population

**Figure 1:**
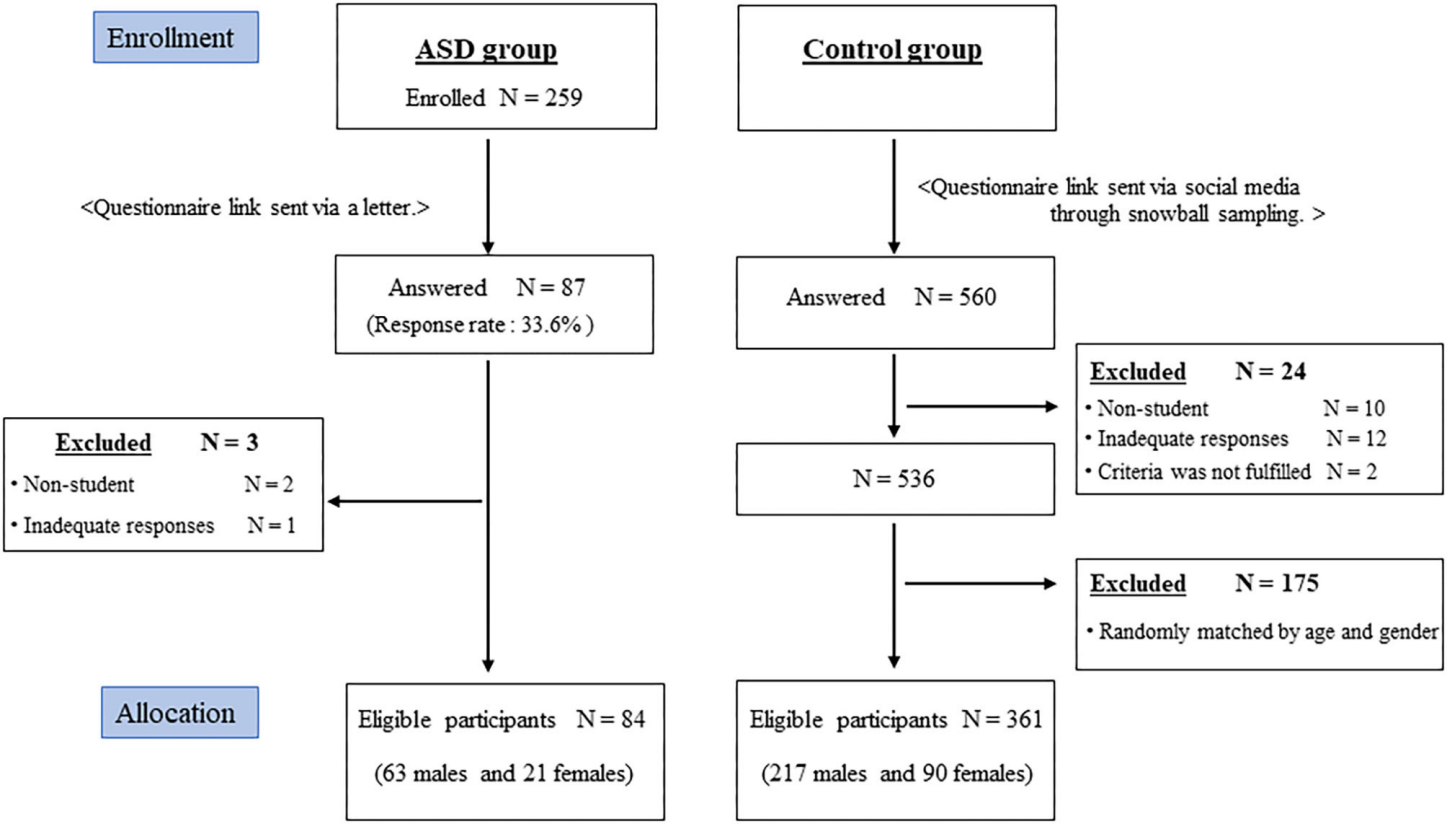
Flow diagram of the process of sampling the autism spectrum disorder group and a matched control group. ASD, autism spectrum disorder.

A flowchart of the recruitment process is depicted in Figure 1. We received responses from 87 participants (response rate: 33.6%). Of these, two were excluded because they did not meet the study criteria and one because of inadequate answers. Thus, there were 84 eligible participants with ASD (63 males and 21 females) who completed this study (Table 1). The mean age in the ASD group was 11.6 ± 3.1 years. Of the ASD group, 42 were in elementary school, 24 in junior high school, and 18 in high school. For the control group, we used data from 560 individuals to whom the same questionnaire was sent. We applied random age and gender matching for the control group. A total of 361 participants (271 males and 90 females) were selected as controls. The mean age in control group was 11.2 ± 3.4 years.

**Table 1:** Characteristics of the participants.

	**ASD**	**Control**
*N*	84	361
**Gender**		
Male, n (%)	63 (75.0)	271 (75.1)
Female, n (%)	21 (25.0)	90 (24.9)
Age	11.6 ± 3.1	11.2 ± 3.4
**School level**		
Elementary school	42	182
Special class	14	0
Special school	6	0
Junior high school	24	107
Special class	10	0
Special school	3	0
High school	18	72
Special school	5	0

*ASD, autism spectrum disorder*.

### Between-Group Differences in Changes in Children's and Parents' Daily Lives Because of COVID-19

Table 2 depicts the percentage of each response and internet or digital media use time in both groups. Most children −76.2% [64/84, 95% confidence interval (CI): 65.7–84.8%] in the ASD group and 77.8% (281/361, 95% CI: 73.2–82.0%) in the control group— were reported to experience stress due to the pandemic. There were no significant differences in the rate of children who were reported to experience stress due to COVID-19 and parents who spent more time playing games with their children between the two groups. Regarding the number of visits to the private agency, there was a significant decrease in the control group (77.6%, 280/361, 95% CI: 72.9–81.8%) as compared to the ASD group (31.0%, 26/84, 95% CI: 21.3–42.0%). The pre-pandemic internet or digital media use time in the ASD group was reported that significantly longer (median [quartile]: 3 h [2–5]) than in the control group (2 h, [1.5–3]) (*p* < 0.001). Internet use time significantly increased after school closure in both the ASD group (*p* < 0.001) and the control group (*p* < 0.001). The digital media use time significantly increased in the control group than in the ASD group. (ASD: one point two 5 h, [0–2], control; 2 h, [1–3]) (*p* = 0.002).

**Table 2:** Comparison of responses between the autism spectrum disorder and control groups.

	**ASD**	**Control**	***P***
*N*	84	361	
Age	11.6 ± 3.1	11.2 ± 3.4	0.419
**School type**			
Elementary school, n (%)	42 (50.0)	182 (50.4)	0.95
Junior high school, n (%)	24 (28.6)	107 (29.6)	
High school, n (%)	18 (21.4)	72 (20.0)	
**Is your child stressed by the COVID-19 pandemic?**			
Yes, n (%)	64 (76.2)	281 (77.8)	0.744
No, n (%)	20 (23.8)	80 (22.2)	
**Parents who spent more time playing games with their children?**			
Yes, n (%)	40 (47.6)	177 (49.0)	0.816
No, n (%)	44 (52.4)	184 (51.0)	
**Is your child making fewer visits to the private agency, for example education centers, and rehabilitation centers?**			
Yes, n (%)	26 (31.0)	280 (77.6)	< 0.001^**^
No, n (%)	58 (69.0)	81 (22.4)	
**Is your child making fewer visits to the after school activities, e.g., lessons, and culture schools?**			
Yes, n (%)	44 (52.4)	184 (51.0)	0.816
No, n (%)	40 (47.6)	177 (49.0)	
**Internet or digital media use time, median hour (quartile)**			
Before pandemic	3 (2–5)	2 (1.5–3)	< 0.001^**^
During pandemic	5 (3–7)	4 (3–6)	0.12
Change time	1.25 (0–2)	2 (1–3)	0.002^**^

*ASD, autism spectrum disorder. The chi-square test was used for categorical variables and the Mann-Whitney U-test for numerical variables*.

** *p < 0.01*.

## Discussion

Our results based on parental report indicated that internet or digital media use time was longer in the ASD group than the control group on weekends before the pandemic and increased in both groups during the pandemic. However, the digital media use time was significantly increased in the control group. To the best of our knowledge, this is the first study clarifying the difference of internet and digital media use time between children and adolescents with ASD and without ASD before and during the COVID-19 pandemic.

Although children have been less severe clinical manifestations and infected rate of COVID−19 than those of adults (10), the psychological effect and the change in their lifestyles is a serious problem. The COVID-19 pandemic has been the cause of mental health problems, public health crises, social isolation, and economic downturns; the cumulative effect may worsen mental health among children and adolescents (11). A study in mainland China during the initial phase of the COVID-19 pandemic reported that more than half of the general public rated the psychological impact as moderate to severe, and about one-third reported moderate to severe anxiety during that phase (12). In particular, students have been reported to be experiencing the psychological impact of the COVID-19 pandemic and higher levels of stress, anxiety, and depression (12). Especially for students, school closure has reduced opportunities for communicating with friends as well as access to school mental health services (13). While online education is a practical and recommended measure during the pandemic (14), at least in our area, it was not adequately serving educational purposes or facilitating communication with friends or teachers. To date, although there have been no consistent results regarding gender differences in children's pathological internet use, many studies show male dominance; moreover, the prevalence of problematic internet use increased with school grade (15). According to our findings, children in control group increase time of internet or digital media use than in the ASD group. This is a surprising finding because adolescents with ASD are considered to be at a higher risk for problematic media use and internet addiction (16). So et al. showed the higher rate of problematic media user in ASD and/or ADHD than in general population evaluated by the rating scale of internet addiction (17). Chen et al. reported that there was an inverse relationship between autism tendency and internet addiction in their school-based and a longitudinal investigation (18). According to meta-analysis, there were no consistent evidence between Internet use and ASD because autistic traits were so widely among individual, though there were moderate association between Internet use and ADHD (19). We evaluated only the digital media use time, in future study it is necessary to examine not only the media use time, but also the association between characteristics of ASD and tendency of internet addiction. Consensus guidance indicated that psychological stress related to the COVID-19 pandemic may contribute to developing a mindset that rationalizes new unhealthy habits, such as engaging in poorly controlled use of the internet or excessive screen time (20). Children might rationalize problematic media use on the grounds of school closure. In any case, psychological stress in children due to school closure affected not only participants with ASD but also those without, and our results indicate that internet use time increased in the control group more than it did in the ASD group. Several researchers have reported that problematic internet use leads to deterioration in mental health, such as the development of depression and anxiety (21, 22). COVID-19-related anxiety was also associated with the severity of problematic internet use (23). Excessive smartphone uses such as seeking information on COVID-19 might have adverse consequences. Protracted periods of isolation, technology-based activity, and limited face-to-face interaction have the danger of solidifying unhealthy lifestyle patterns, intensifying technology-related disorders, and leading to difficulties in re-adaptation when the COVID-19 crisis has passed (24). Children have experienced at least 2 months of school closure, and in this period, school authorities have been rethinking or considering terminating events such as physical education, club activities, and school trips in accordance with infection control measures. From the above, it can be inferred the school during pandemic is so boring for children.

The current study has several limitations. First, the recruitment methods for the ASD and control groups differed; the ASD group was invited to participate by mail and the control group through snowball sampling. The ASD group were intended all patients who met the criteria, however a selection bias was unknown due to the sampling methods. As snowball sampling was not based on a random selection, the study population might not be representative of the general population. Second, our study relied on parent reports, and did not collect personal information, such as the domestic environment, including economic status, level of intelligence in children, and level of education in mothers, because of ethical requirements concerning anonymity and confidentiality. Therefore, the possibility of information bias cannot be disregarded. Third, this study did not indicate the way mothers grasp their children's media time. Depending on the background and characteristics of the children, it may be difficult for parents to grasp their children's media use time exactly. Forth, our assessment did not include detailed characteristics of the ASD group. There are individual differences in the characteristics of ASD, which might affect internet use. Fifth, as the participants belonged to a single prefecture, attempts to generalize our results to other prefectures must be undertaken with caution. Sixth, the present study employed a cross-sectional design. Further prospective studies should be performed on the same group of participants over a longer period.

## Conclusion

Our study makes a valuable comparison of internet use time between children and adolescents with and without ASD before and after school closure related to the COVID-19 pandemic. Following school closure, increased internet and digital media use time was observed in most children. It is necessary to formulate strategies to prevent excessive internet use in the post-pandemic world, wherein children's school and daily lives will no longer be the same.

## Data Availability Statement

The raw data supporting the conclusions of this article will be made available by the authors, without undue reservation.

## Ethics Statement

This study was approved by the concerned institutional review board (IRB No. 2006014). Written informed consent to participate in this study was provided by the participants' legal guardian/next of kin.

## Author Contributions

KK conceived, designed, managed the study, and wrote the manuscript. RH, KN, and AY collected the data. RH and KN performed the data analysis FH and SU supervised to study design, and revision of the manuscript. All authors provided critical feedback, contributed to the final manuscript, and agreed to the publication of the article.

## Conflict of Interest

The authors declare that the research was conducted in the absence of any commercial or financial relationships that could be construed as a potential conflict of interest.
